# Electromagnetic induction properties of filamentous bacteria in sediment

**DOI:** 10.1093/pnasnexus/pgaf011

**Published:** 2025-01-18

**Authors:** Fuxing Kang, Robin Bonné, Lars Peter Nielsen

**Affiliations:** College of Resources and Environmental Sciences, Nanjing Agricultural University, 210095 Jiangsu, China; Center for Electromicrobiology, Department of Biology, Aarhus University, 8000 Aarhus C, Denmark; Center for Electromicrobiology, Department of Biology, Aarhus University, 8000 Aarhus C, Denmark; Center for Electromicrobiology, Department of Biology, Aarhus University, 8000 Aarhus C, Denmark

**Keywords:** bioelectromagnetic induction, spatial electric oscillation, cable bacteria, square wave, inductive spatial boundary

## Abstract

Microbial perception of spatial electromagnetic fields is essential for navigation and communication on Earth's surface system, but current understanding of this phenomenon is limited. At present, cable bacteria of the Desulfobulbaceae family have the longest known range of electron transport. In fact, the flow of electrons along these long filamentous bacteria generates an external electrostatic field, suggesting a potential for electromagnetic induction mirroring that of metallic wires. In this study, we measured the responses of cable bacteria to externally applied electric waves. We noted the formation and disappearance of square waves caused by a pair of spatially variable electric fields, generating negative and positive mirror-symmetric inductions (±1.20 mV in marine sediment) along the horizontally filamentous bacterial layer. Both seawater *Candidatus* Electrothrix and freshwater *Ca.* Electronema exhibited this electric induction. The distinct spatial boundary of bacterial induction was strictly confined within 12.5 mm below the surface of the seawater sediment. The results of this study open further avenues of research into understanding how bacteria sense and respond to spatial electromagnetic information.

## Introduction

The ability of microorganisms to perceive electromagnetic fields is crucial for their navigation and communication on Earth's surface. However, our current understanding of this process is limited to 2 specific scenarios: magnetotactic bacteria that use extracellular magnetite (1–6) to detect the Earth's magnetic field (2, 3, 7–9) and ion channels in microbial membranes that generate potentials in response to electric stimuli (10–13). Evidence in the literature has been sparse concerning conductive filamentous bacterial structures analogous to metal wires. These structures may actually be capable of inducing significant spatial electromagnetic fluctuations. In this study, we confirmed that cable bacteria, which grow widely in both marine and freshwater sediments, can sense electromagnetic oscillations. This evidence may provide new perspectives regarding the sensing of wireless electromagnetic information by microorganisms (14–17).

The first report of cable bacteria came from Nielsen et al. in 2010 (18), and cable bacteria have been defined as a group of filamentous bacteria from the Desulfobulbaceae family that are capable of centimeter-long electron transport (19). By coupling the oxidation of H_2_S (20) or acetate (21) in deeper sediment to the reduction of O_2,_ NO_3_^−^, or Fe^3+^ at the surface (21–28), electrons can be transported over conductive periplasmic fibers to form an internal circuit used to equilibrate external reactions (29). The conductive fibers in cable bacteria extend over the whole filament of up to 10,000 cells, as an interconnected electrical network with conductivities of >10 S·cm^−1^ and electron mobilities in the range of 0.1 cm^2^·V^−1^·s^−1^ (30). Their highly linear current-voltage characteristics, as well as their resistive impedance characteristics, indicate that cable bacteria behave as classical conductive nanowires (22, 30). Efforts to understand the function of electron transport in cable bacteria have identified roles related to physiological adaptation (31) and elemental geochemistry (32–35). Nevertheless, whether this electron-conducting system supports the type of electrical induction similar to that present in inorganic metal wires remains unclear. Therefore, this type of biological induction may present new evidence concerning biological communication beyond the known applications related to physiological adaptation and elemental geochemistry in cable bacteria (36).

All conductors that rely on the directional motion of electrons are theoretically capable of electromagnetic induction. The spatial static electric field caused by electrons flowing along filamentous cable bacteria has been measured previously using electrostatic potential (EP) microsensors and has been reported to increase by ∼0.6 mV within 1.4 cm below the surface of the sediment (17, 37, 38). Evidence from conductivity tests and electrostatic field measurements around cable bacteria led us to hypothesize that cable bacteria respond to electromagnetic oscillations similarly to inorganic metal wires. In the present study, we successfully enriched and cultured a marine strain of *Candidatus* Electrothrix and a freshwater species of *Ca.* Electronema bacteria, developed a set of new sensor/electrodes, and employed them to meticulously measure the electromagnetic induction responses of filamentous cable bacteria to spatial electromagnetic fluctuations.

## Results

### Induction in a layer of cable bacteria by an electric field

**Fig. 1.**
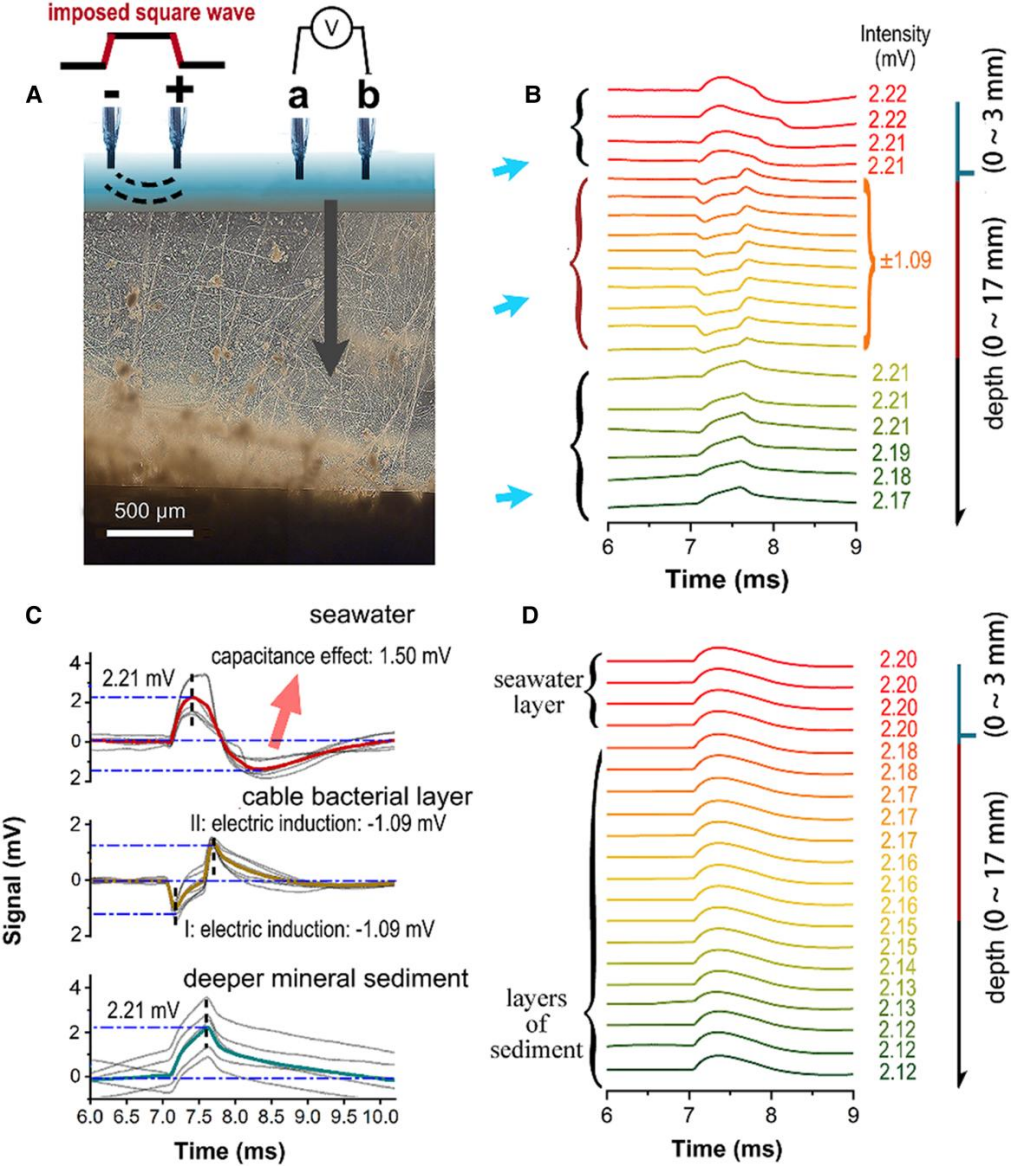
Induction in the cable bacteria layer by our imposed square wave. (A) Electrical induction in filamentous cable bacteria caused by a square wave emitted nearby. The 2 tips of the emitting electrode immersed in seawater (top left) emitted a square wave (5.0 V, 0.5 ms), producing a deterministically varying spatial electric field that could be detected by the receiving electrode (a and b). (B) Changes in the induction signals (depth indicated by colors transitioning from red to dark green) as the depth increased from 4.0 mm above the sediment to 17.0 mm below its surface, with readings taken every millimeter. Reported data comprise the average values of 3 replicate experiments. (C) Characteristics of the seawater, induction in the cable bacteria layer, and deep mineral sediment signals. All data represent averages of 5 replicate readings (gray plots). (D) Signals obtained in the control experiment (depth is indicated by colors transitioning from red to dark green, with readings taken every millimeter). Reported data represent the average of 3 replicate readings.

To examine electrical induction in cable bacteria, we collected marine sediment cores with abundant cable bacteria on the sediment surface from Kalø Vig, Denmark. A double-platinum-needle electrode emitting a square-shaped radio wave radio (emitting electrode) was placed in the overlying seawater 6 cm from the sediment's surface, and the induced signal was monitored using another double-platinum-needle electrode (points a and b in Fig. 1A detailing the receiving electrode; Extended Data Figs. S1–S3). If the cable bacterial layer experienced induction from the emitting electrode, a corresponding signal would theoretically be measurable between tips a and b of the receiving electrode (Fig. 1A).

As expected, with a square wave at +5.0 V and a 0.5 ms width, we observed 3 different signal patterns in the seawater, surface sediment (i.e. cable bacterial layer), and deeper sediment (i.e. minerals but with no cable bacteria layers) (Fig. 1C). In the seawater, there were a group of signals; in the deeper mineral sediment layer (with no cable bacteria), there was only one positive peak, at ∼+2.17 to 2.21 mV. These results were attributed to the capacitor effect from the seawater and sedimentary minerals present in the respective layers. Notably, in the middle layer containing the cable bacteria—classified as *Ca.* Electrothrix aarhusiensis MCF4-14 (marked in green in Fig. 3D)—we observed a pair of signals with opposite directions and almost equal amplitudes, at ±1.09 mV. For ease of observation, we amplified these signals and transposed them to 7.2 and 7.7 ms, as is detailed in the middle portion of Fig. 1C. After the cable bacterial layer was removed from the sediment, both induction signals disappeared and returned to the same levels observed in the seawater and deeper mineral sediment layers as a result of the capacitor effect (Fig. 1D). These results indicated the presence of an electrical induction signal in the cable bacterial layer that differed significantly from that in the water and mineral layers.

**Fig. 2.**
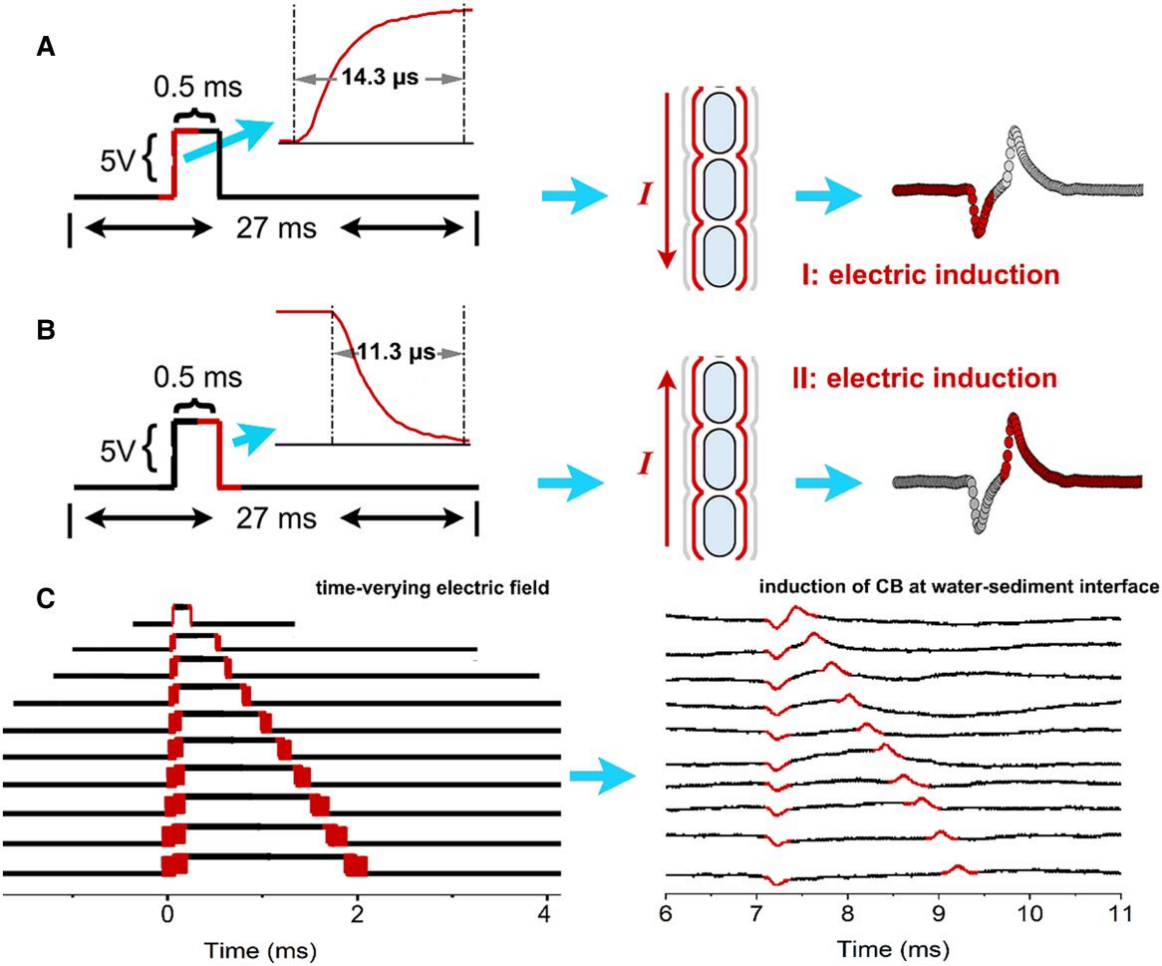
Relationship between induction signals and emitted square-wave signals. A periodic square-wave electric potential (+5 V for 0.5 ms) was applied between 2 tips (spaced 0.5 cm apart) of the emitting electrode to produce a fluctuating electric field. Induction signals were then measured in the cable bacteria (CB) layer at 6 ± 1.0 mm below the surface of the seawater sediment. (A) An increase in the square-wave voltage was accompanied by the appearance of a corresponding negative induction signal in the CB layer. (B) A decrease in the square-wave voltage was accompanied by a corresponding positive induction signal in the CB layer. The directions of the electric fields induced in the filamentous CB (*Ca.* Electrothrix aarhusiensis MCF4-14, middle portions of panels A and B) were opposite in charge to the signals emitted by the emitting electrode. The red curves in the cellular periplasm represent the conductive fibers of the CB that are capable of sensing changing electric fields. (C) Square-wave voltages of varying periods (amplitude: 5.0 V; periods ranging between 0.2 and 2.0 ms in 0.2-ms increments) emitted from the two tips (spaced 0.5 cm apart) of the emitting electrode produced a varying electric field stimulus. The same time intervals were observed between the negative and positive peaks in the induced signal in the CB layer, with a correlation coefficient of *R*^2^ = 0.9999. The experiment was performed with a CB density of 42 to 46/mm^2^. The data reported in panels C and D represent the average of 3 replicate readings.

The generation of electric induction in metal conductors depends on spatial fluctuating electric fields (39, 40), based on the law of electromagnetic induction (41). We confirmed that the electric induction we observed in our layer of the cable bacteria also followed the same physical law, as the temporal lag between different recorded induction signals correlated consistently with the square-wave electric field pulses we sent through the emitting electrode. When the spatial square wave jumped from 0 to +5 V over 14.3 µs (Fig. 2A; Extended Data Fig. S4), a negative induction signal that opposed the time-varying electric field occurred in the cable bacteria layer (Fig. 2A, right). When the spatial square wave returned from +5 to 0 V over 11.3 µs (Fig. 2B; Extended Data Fig. S4), a positive induction signal that similarly opposed the time-varying electric field was also recorded in the cable bacteria layer (Fig. 2B, right). This pair of induction signals in the cable bacteria layer was only generated in response to changes in the applied square wave.

According to these properties, we noted that the temporal spacing between 2 induced signals always equaled the period of the applied wave. Thus, when we increased the widths of the spatial square waves from 0.2 to 2.0 ms in 0.2-ms increments (Fig. 2C, left), the temporal differences between the negative and positive induction signals in the cable bacteria layer varied accordingly (Fig. 2C, right). A robust good linear correlation between the spatial wave widths and the measured differences in time between the induced signals (slope = 1.00 in Extended Data Fig. S5; *R*^2^ = 0.9999), indicated that the measured induction signals were always generated by the electromagnetic oscillation of the square waves we sent through the emitting electrode. In addition, when the positive and negative tips of the emitting electrode were switched, we noted a reversal in the induction signal (Extended Data Fig. S6). Taken together, these results demonstrate that the induction signals we observed in the cable bacteria layer were caused by our oscillating spatial electric field.

### Types of cable bacteria responsible for electric induction

**Fig. 3.**
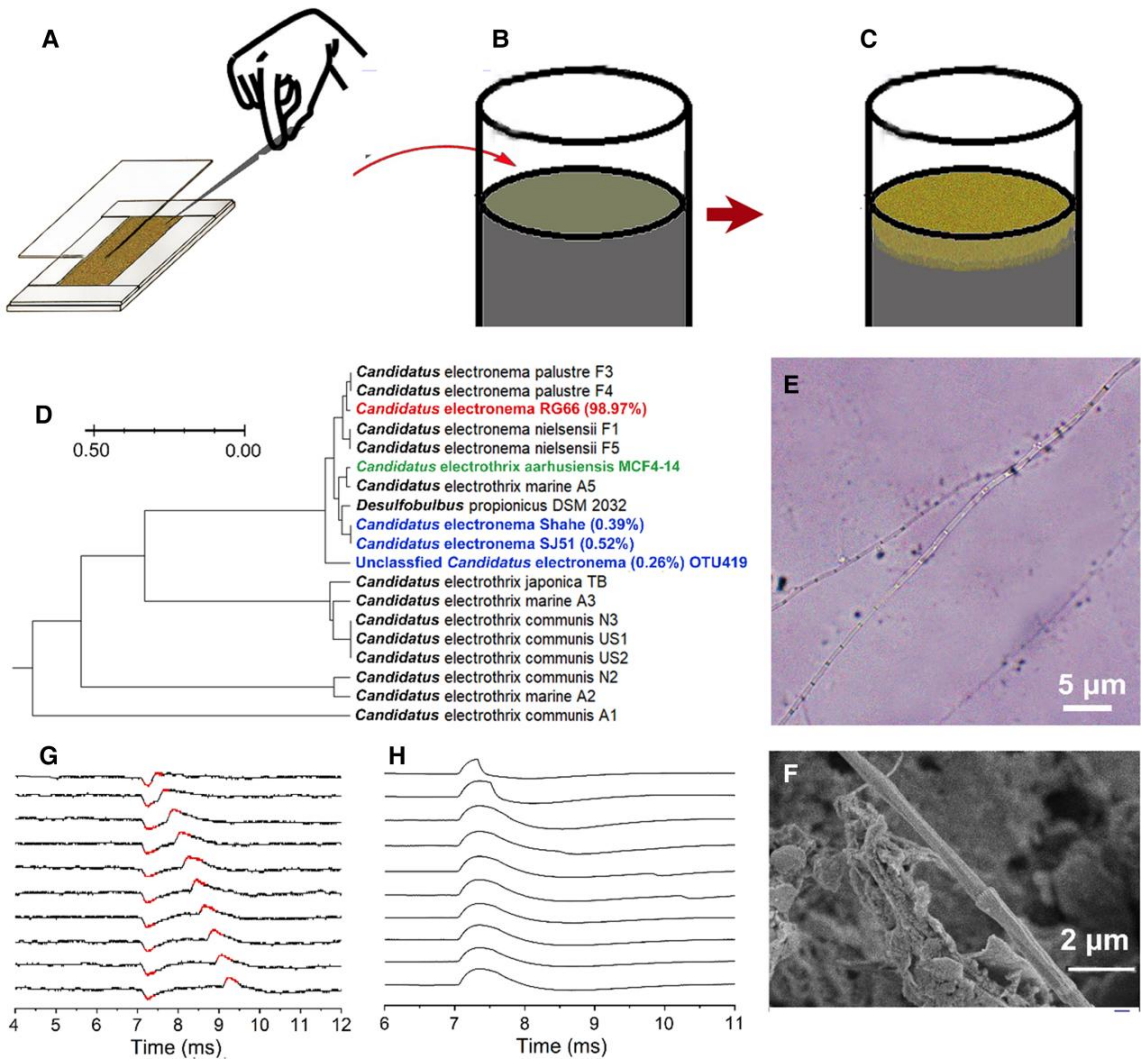
Species of cable bacteria capable of bioelectric induction. (A) Cable bacteria grown in a sediment substrate on a microscope slide. A single cable bacterium filament was picked using a glass hook and (B) transferred to an autoclaved sediment substrate rich in black organic matter (sediment core in an acrylic cylinder 7 cm in diameter). (C) A single cable bacterium filament was incubated for 3 weeks at 15 °C until a layer of cable bacteria was formed in the upper portion of the sediment. (D) Phylogenetic affiliation of the successfully cultured *Ca.* Electronema sp. RG66 (red) with other Electrothrix and Electronema identified previously (based on 16S rRNA gene sequencing). *Ca.* Electronema shehe, *Ca.* Electronema SJ51, and unclassified Electronema OTU419 (blue font) were identified in freshwater sediment. *Ca.* Electrothrix aarhusiensis MCF4-14 (green font) represents the main cable bacterium used in the experiments detailed in Fig. 1. (E) Optical micrograph and (F) scanning electron micrograph of *Ca.* Electronema RG66. (G) Negative and positive induction signals produced by *Ca.* Electronema RG66 in the sediment substrate, with square waves of 5.0 V amplitude and periods ranging between 0.2 and 2.0 ms in 0.2-ms increments. Reported data represent the average value of 3 replicate readings. (H) Sediment with no *Ca.* Electronema RG66 (control) showed only one signal, corresponding to the capacitor effect of the sediment material with no discernable induction signal. Reported data represent the average value of 3 replicate readings.

The most predominant species of cable bacteria in seawater sediments and coastal zones (Figs. 1 and 2) are *Ca.* Electrothrix aarhusiensis MCF4-14. These were also the bacteria primarily responsible for the induction we observed in response to our oscillating electric field; however, to explore the full range of bacteria that exhibit this phenomenon, we also isolated and grew a freshwater cable bacterium in an autoclaved sediment substrate, based on the experimental procedure outlined in Fig. 3A–C (with further details presented in the Supplementary Information). The dominant strain of cable bacteria in this sample was classified genetically as freshwater *Ca.* Electronema RG66 (abundance, 98.97%) (Fig. 3D), and exhibited the same microscopic morphology noted in our previous observations (Fig. 3E and F). We also noted several minor populations of other cable bacteria (*Ca.* Electronema shehe, SJ51, and OTU419; combined abundance, 1.03%) in our cultured sediment.

We used the same method to characterize the electrical induction properties of *Ca.* Electronema RG66. As was expected, the temporal lag between the two induction signals that we observed was identical to the 0.5-ms width of the square wave that we sent through the emitting electrode (Fig. 3G; Extended Data Fig. S4). When the widths of the square waves were again varied between 0.2 and 2.0 ms in 0.2-ms increments, the differences in time between the negative and positive induction signals measured in the cable bacteria culture corresponded to the respective widths of the applied square waves (Fig. 3G). This mirrored the results we obtained in the seawater sediment (*Ca.* Electrothrix aarhusiensis MCF4-14), confirming that this type of electric induction can occur in both saltwater and freshwater cable bacteria.

### Boundary of electrical induction in the seawater sediment

**Fig. 4.**
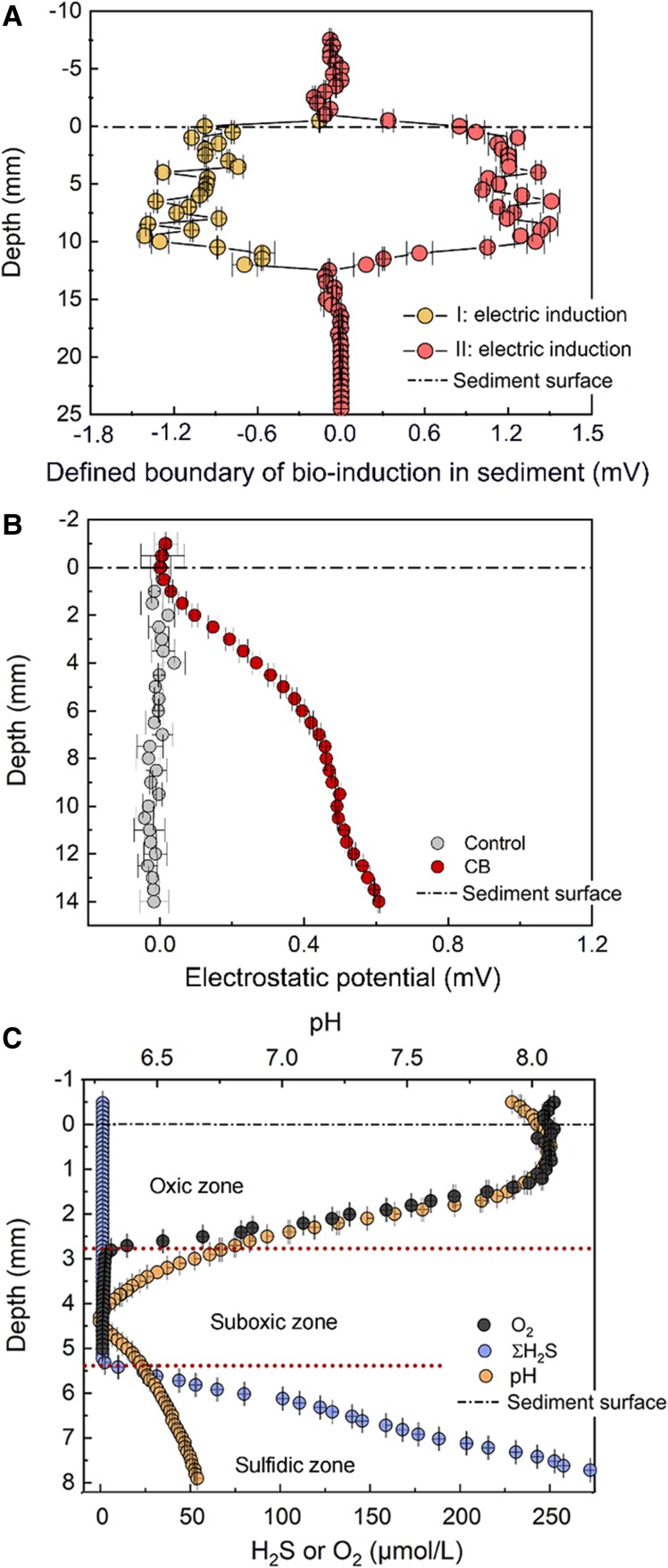
Boundary of bioelectric induction in marine sediment. (A) Electric induction profiles of the cable bacteria (CB) layer at increasing depths. The data were collected from the negative signal at 7.2 ms and positive signal at 7.7 ms, detailed in the middle portion of Fig. 1C, as the depth increased. Each dataset was acquired at every 500-μm increase in depth. Error bars represent the variations in 3 replicate readings at each measured depth. (B) Electrostatic field in the sediment measured using an EP microsensor (38). The potentials read by this microsensor relative to an Ag/AgCl reference electrode in seawater were defined as the zero potential for the purpose of making comparisons between the EP values in sediment samples without (gray: control sample with no CB) and with CB (red). Each dataset was acquired at depths that increased in 100-μm increments. Error bars represent the standard deviations of triplicate measurements. (C) Oxygen, sulfide, and pH profiles produced by the CB. Error bars represent the standard deviations of triplicate measurements. Each dataset was acquired at depths that increased in 100-μm increments.

We then proceeded to explore the boundaries of the electrical induction signals in the saltwater cable bacteria. The induction potentials were noted to be present in the range of 12.5 mm below the water-sediment interface (Fig. 4A), at an average potential of approximately ±1.20 ± 0.10 mV. The amplitudes of the positive and negative signals were symmetrical, confirming that they resulted from our applied square waves.

We then compared this boundary to that of cable bacteria dependent on the electrogenic sulfur oxidation (*e-SO_X_*) (42, 43). Fine-scale measurements of the EP (38, 44, 45) in these sediments showed that the predicted electric field outlined the sphere of the cable bacteria themselves (44). In these experiments, EP referred to the electric potential between the top and bottom generated by the reduction-oxidation reaction of H_2_S and O_2_, and the changing electric field referred to the electromagnetic induction generated by the cable bacteria in response to external electromagnetic oscillation. Because the two circuits were not superimposed, they represented two independent closed circuits. Therefore, EP indicated only the boundary of the cable bacteria activity.

As these cable bacteria coupled H_2_S oxidation (21) to O_2_ reduction (21–26), the sphere of influence controlled by this *e-SOx* process spanned from the oxic zone to the suboxic and sulfidic zones (Fig. 4B), resulting in distinct pH, O_2_, and H_2_S profiles in the sediment. However, when measured in conjunction with EP, these traditional parameters were estimated to occur within 0.5 mm below the sediment's surface (Fig. 4C) and thus could not accurately indicate the lower limit of the cable bacteria's sphere of activity in the sulfidic zone. Thus, the actual boundary of the cable bacteria activity as determined through our induction experiments was deeper than what we measured based on the *e-SOx* (pH, O_2_, and H_2_S) profiles (18, 43). The bio-induction signal occurred in the range of ∼12.5 mm below the seawater-sediment interface.

## Discussion

### Comparison with magnetotactic bacteria

Our findings demonstrated that the electron-conducting system in cable bacteria facilitates electromagnetic induction that mirrors the well-known phenomenon similarly in inorganic metallic conductors. This ability to sense electromagnetic fields was also reminiscent of a similar property in magnetotactic bacteria, which rely on both intra- and extracellular magnetite (1–5) to sense the Earth's magnetic field (2, 3, 7, 8). However, in this study, we did not observe magnetite in or near our cable bacteria.

Furthermore, an additional potential scenario can likewise be eliminated. It has been previously reported that iron exists in layers of cable bacteria, mainly as the ferrous (II) ion, at concentrations far lower (<50 μmol Fe^2+^·kg^−1^) than the values found in deeper sediment layers (∼70–150 μmol·kg^−1^ at a depth of 4–6 cm the sediment surface) (46). As a result, the electric induction observed in this study was only found to occur in the cable bacteria layer, and was not present in the iron-rich layer of the deeper sediment, excluding the possibility of electric induction occurring in the major iron species in the sediments. Our results therefore suggest that the induction signals that occurred in the cable bacteria layer were independent of the metal-containing minerals present in the sediment and represent a novel form of bioelectric induction caused by an interaction between the filamentous cable bacteria and our oscillating electromagnetic field.

### Equivalent model of electric induction

To characterize this form of bioelectric induction, we constructed an equivalent model for electrical induction (Extended Data Fig. S7). According to the right-hand rule in electromagnetism, a periodic square-wave potential with a high rate of fluctuation (+0.350 V·μs^−1^) induces a magnetic field around the line connecting the poles of the electrode carrying the signal. In the fluctuating magnetic field produced by our emitting electrode, the filamentous bacterial conductor produced a negative electrical signal based on Lenz's law (Extended Data Fig. S7, right). Similarly, a periodic square-wave potential with a low rate of fluctuation (−0.442 V·μs^−1^) also induced a magnetic field, generating a positive electrical signal in the filamentous bacteria (Extended Data Fig. S7, right). This model thus accurately explains how the two induction signals in our layer of cable bacteria were triggered by our oscillating electric fields.

We further simplified this model by replacing the layer of filamentous cable bacteria with a bare conductive copper wire (Extended Data Fig. S7C). We observed that the time lags between the two induction signals matched the square-wave widths of the spatial square waves (0.2, 0.4, 0.6, 0.8, 1.0, 1.2, 1.4, 1.6, 1.8, and 2.0 ms) (Fig. 2C). The electromagnetic induction patterns in the copper wire (Extended Data Fig. S7D) matched those noted in the cable bacteria layer (Fig. 2C). This result further confirmed that cable bacteria can react to external electric fields similarly to inorganic metallic conductors, following the laws of electromagnetic induction (41).

### Vertical component of bioelectrical induction in the cable bacteria layer

According to our proposed equivalent model, we hypothesized that no induction signal would be measurable between the upper and lower layers of cable bacteria as the electric field was moved along the horizontal axis (Fig. 1A) because the induced signals would have been oriented horizontally, according to Lenz's law and the right-hand rule (41). This hypothesis was tested by measuring the vertical component of the induced signals in the cable bacteria layer. We used a receiving electrode with 2 bare platinum tips spaced 0.5 cm apart to detect the vertical component, using the same square-wave source detailed in Fig. 1A. Our results (Extended Data Fig. S8) revealed that when both tips of the receiving electrode were submerged either in the seawater (case 1 in Extended Data Fig. S8B and C) or the mineral sediment without cable bacteria (case 3 in Extended Data Fig. S8B and C), only the background signal from the square waves (7.2 ms) and the capacitance effect were detected. Conversely, when the two tips were embedded in the cable bacteria layer, a small and sharp negative induction signal at 7.2 mV was superimposed near the background signal, but no positive induction signal was found. Theoretically, this weak superimposed induction signal was hypothesized to vanish if the line between the receiving electrode tips was perpendicular to the line between the tips of the emitting electrode. However, this ideal geometric arrangement was not feasible in practice, and the slightly superimposed signal could not be completely eliminated without the persistence of a geometric transverse spacing component between two tips of the receiving electrode. Nevertheless, the absence of a pair of opposite and equal induction signals suggests that the differences in the induction signals between the upper and lower layers of the cable bacteria were negligible.

### Potential biological-geological-environmental implications

The presence of this bioelectric induction phenomenon in both sea- and freshwater sediments may carry a substantial geoenvironmental significance. It indicates that electromagnetic oscillations may be detectable by the filamentous “antennas” of cable bacteria communities, broadcasting the information through biofilms or biological mats in and on sediments, allowing the bacterial populations to perceive spatial electromagnetic information. Additionally, similar electronic signaling structures may also be present in certain migratory animals such as pigeons and cetaceans, allowing them to travel to precise locations worldwide (14–17, 47) as the motions of their conductive fibers relative to the generally static magnetic field of the Earth likely mirror the electromagnetic oscillations we used in our experiments. The results of this study may therefore generate other general insights that extend beyond the field of bacterial microbiology as well.

## Supplementary Material

pgaf011_Supplementary_Data

## Data Availability

All data are included in the manuscript and Supporting Appendix. Measurement of electric induction in cable bacterial layer, preparation of the emitting electrode and receiving electrode, and collection and incubation of cable bacteria is included in the Supporting Appendix. Both the structural feature of the emitting electrode and receiving electrode and the measurement detail for the electromagnetic induction of filamentous bacteria is included in the Supporting Appendix.
