# Supplementary material for: Electromagnetic induction properties of filamentous bacteria in sediment
Source: PNAS Nexus. 2025 Jan 18;4(2):pgaf011. doi: 10.1093/pnasnexus/pgaf011 (PMC11787994; doi:10.1093/pnasnexus/pgaf011)
Supplement: pgaf011_Supplementary_Data [file pgaf011_supplementary_data.zip › PNASNEXUS-PNASNEXUS-2024-00414RR-s02.docx]

**Electromagnetic induction properties of filamentous bacteria in sediment**

Fuxing Kang^a, b, *^, Robin Bonné^b^, and Lars Peter Nielsen^b, *^

*^a^* College of Resources and Environmental Sciences, Nanjing Agricultural University, 210095 Jiangsu, China

*^b^* Center for Electromicrobiology, Department of Biology, Aarhus University, 8000 Aarhus C, Denmark

To whom correspondence may be addressed (*): lpn@bio.au.dk or kang@njau.edu.cn

Manuscript prepared for ***PNAs Nexus***

January 3th, 2025

**Materials and Methods**

Appendix including the extended data Figs. 1-8 is available in the online version of the paper

**Collection of sediment.** The upper 25 cm of marine surface sediment (inner diameter: 50 mm) below 0.5-m depth seawater was collected in April 2021 using a column corer from Kalø Vig, Denmark (11°C, 56°16' 57.58" N, 10°27'17.54" E). The sediment columns were submerged in a seawater aquarium (15.2 °C; 25‰ Red Sea salts) and incubated with bubble aeration. The incubation time was from April to July for 90 days. During this long-term incubation process, the layering of cable bacteria would be particularly noticeable. We carried out this study when a dense layer of cable bacteria grew well on surface sediment ((42–46) per square millimeter under microscopic examination).

**Electric induction in cable bacterial layer.** An oscillating electric field 4-6 cm from the sedimentary surface was emitted from two tips of a double-platinum-needle electrode (emitting electrode, Extended Data Fig. 1). Specifically, a square-wave potential was applied between two tips (0.5-cm spacing) of the emitting electrode (square wave amplitude: +5.0 V for 0.5 ms; interval between two amplitudes: 27 ms; exposed Pt tip: 300 μm; Pt wire diameter: 50 μm) (Extended Data Fig. 2 and 3). Another double-platinum-needle electrode with a structure similar to that of the emitting electrode was used to detect the induction signals in cable bacteria (receiving electrode). The induction signals in the cable bacterial layer, manipulated by a stepping motor (VT-80, Unisense, Denmark), were received as the depth increased. In addition, based on the same method as the experimental procedure above, a control experiment was performed to examine the possibility of induction after the removal of cable bacteria network on sediment. In brief, a small plastic spoon was used to gently remove the layer of cable bacteria from the sediment surface, followed by a microscopic examination (CX43, Olympus, Japan) to determine that the sediment was free of cable bacteria.

The generation of square-wave pulses and reception of electric signals were performed using a biological signal acquisition and processing system (MedLab®-U/4C501H, China). The instrument was designed to measure the electric stimulation and transmission rate of signals in the nervous system. Therefore, we believe that there are many instruments on the market that can meet the needs of reappearing our experimental results. Signals of less than 3000 Hz were received, but the notching at 50 Hz must be filtered out through a hardware circuit. The position of the emitting electrode relative to the receiving electrode was shown in the Extended Data Fig. 1. Unless otherwise specified, the density of the cable bacteria for the test of bioinduction is (42–46) per square millimeter (under microscopic examination). This calculation was based on the filamentous count of cable bacteria crossing the water/agar-sediment interface under the two-dimensional field of view under an optical microscope.

**Incubation of freshwater cable bacteria.**

The top of 0-20 cm sediment was collected with a grab sediment sampler (Petersen, Wildco, USA) from the Qinhuai River, Nanjing, China, during the autumn season. The sediment passed through a sieve mesh with a 1.00-mm aperture and then filled into an acrylic tube with a 6-cm inside diameter and 12-cm length to yield an 8-cm length of the sedimentary core. A port at the bottom of the sediment core was sealed with a rubber plug, and the sediment core was placed vertically in an aquarium glass tank (35 × 22 × 20 cm). The freshwater from Qinhuai River, after the filtration through a 0.45-μm membrane, was added to the aquarium glass tank. The water surface was higher than the sediment column by 5-8 cm. The air was continuously pumped into the water at 10 L/min to maintain a conducive environment for the growth of cable bacteria. After the cable, bacteria naturally grew over 2 weeks at a 15 ℃ temperature.

A glass hook carefully selected the upper layer of the sediment within 0-3.0 cm, where the cable bacteria were expected to thrive. The individual cable bacterium filaments picked by the glass hook were transferred to the autoclaved sedimentary core (120 ℃ for 30 min) for another growth at 15 ℃ for 2-3 weeks. Afterward, the electric induction of freshwater cable bacteria was tested using the same research methods as the above.

**Identification of cable bacterial strains.**

The cable bacteria strains from seawater and freshwater sediments were identified by 16S rRNA gene amplicon sequencing. In brief, DNA was extracted from two sediment cores 35 days after a cable bacteria transfer using the DNeasy PowerLyzer PowerSoil Kit (Qiagen). The V3-V4 region was PCR amplified using the primers Bac341F and Bac805R(1) and sequenced on an Illumina MiSeq instrument. The resulting sequences were analyzed using the DADA2 pipeline(2); for details, see Scholz *et al*(3).

**pH, O_2_, H_2_S, and EP profiles.** The pH, H_2_S (4), and O_2_ (5) profiles in sediment were collected based on previous studies(4-7) (Unisense, Aarhus, Denmark). The electrostatic potential (EP) of cable bacteria in sediment was measured according to previous studies(8, 9). Results presented in Figures 1 and 4 of paper were from the same sedimentary core.

**References**

1. Herlemann DPR*, et al.* (2011) Transitions in bacterial communities along the 2000 km salinity gradient of the Baltic Sea. *ISME J* 5(10):1571–1579.

2. Callahan BJ*, et al.* (2016) DADA2: High-resolution sample inference from Illumina amplicon data. *Nature Methods* 13(7):581–583.

3. Scholz VV*, et al.* (2021) Cable bacteria at oxygen-releasing roots of aquatic plants: a widespread and diverse plant-microbe association. *New Phytol* 232(5):2138–2151.

4. Jeroschewski P, Steuckart C, & Kühl M (1996) An amperometric microsensor for the determination of H_2_S in aquatic environments. *Anal Chem* 68(24):4351–4357.

5. Revsbech NP (1989) An oxygen microsensor with a guard cathode. *Limnol Oceanogr* 34(2):474–478.

6. Sayama M, Risgaard-Petersen N, Nielsen LP, Fossing H, & Christensen PB (2005) Impact of bacterial NO_3_^-^ transport on sediment biogeochemistry. *Appl Environ Microb* 71(11):7575–7577.

7. Revsbech NP & Jørgensen BB (1986) Microelectrodes: their use in microbial ecology. *Adv Microb Ecol*:293–352.

8. Damgaard LR, Risgaard-Petersen N, & Nielsen LP (2014) Electric potential microelectrode for studies of electrobiogeophysics. *J Geophys Res-Biogeo* 119(9):1906–1917.

9. Meysman FJ (2018) Cable bacteria take a new breath using long-distance electricity. *Trends Microbiol* 26(5):411–422.
