# Supplementary material for: Electromagnetic induction properties of filamentous bacteria in sediment
Source: PNAS Nexus. 2025 Jan 18;4(2):pgaf011. doi: 10.1093/pnasnexus/pgaf011 (PMC11787994; doi:10.1093/pnasnexus/pgaf011)
Supplement: pgaf011_Supplementary_Data [file pgaf011_supplementary_data.zip › PNASNEXUS-PNASNEXUS-2024-00414RR-s01.docx]

**Supporting Appendix**

**Figure legends**

**Extended Data Fig. S1.** Measuring device and relative position of electrodes in the present study.

**Extended Data Fig. S2**. Emitting electrode and receiving electrode for this study.

**Extended Data Fig. S3.** The naked Pt tips of the emitting electrode and receiving electrode. **Extended Data Fig. S4**. Time-varying electric field stimulus with a square wave between two naked Pt tips.

**Extended Data Fig. S5**. Relationship between the width of square waves and time interval of induction signals in cable bacterial layer.

**Extended Data Fig. S6**. Square waveform voltage from -5.0 to 0 V vs. induction signals in cable bacterial layer.

**Extended Data Fig. S7.** The proposed equivalent model for electromagnetic induction in filamentous cable bacteria.

**Extended Data Fig. S8.** Vertical component of bio-induction in cable bacterial layer of sediment.

**
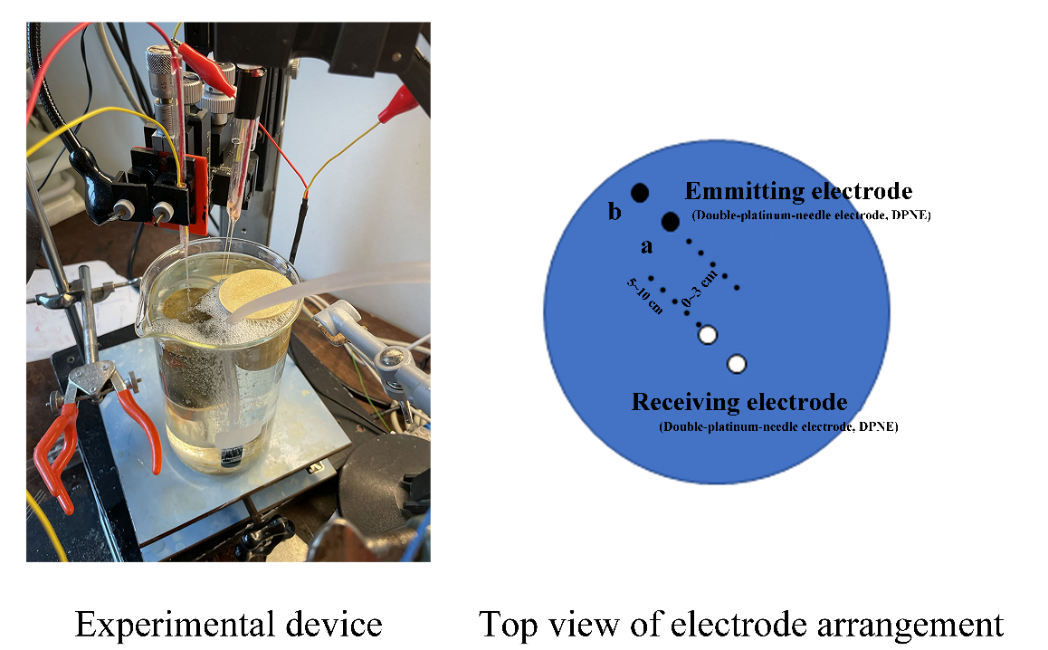
**

**Extended Data Fig. S1.** Measuring device and relative position of electrodes in the present study. The detailed description for this measured device could be found in the Electric induction in cable bacterial layer of Supplementary Information.

**
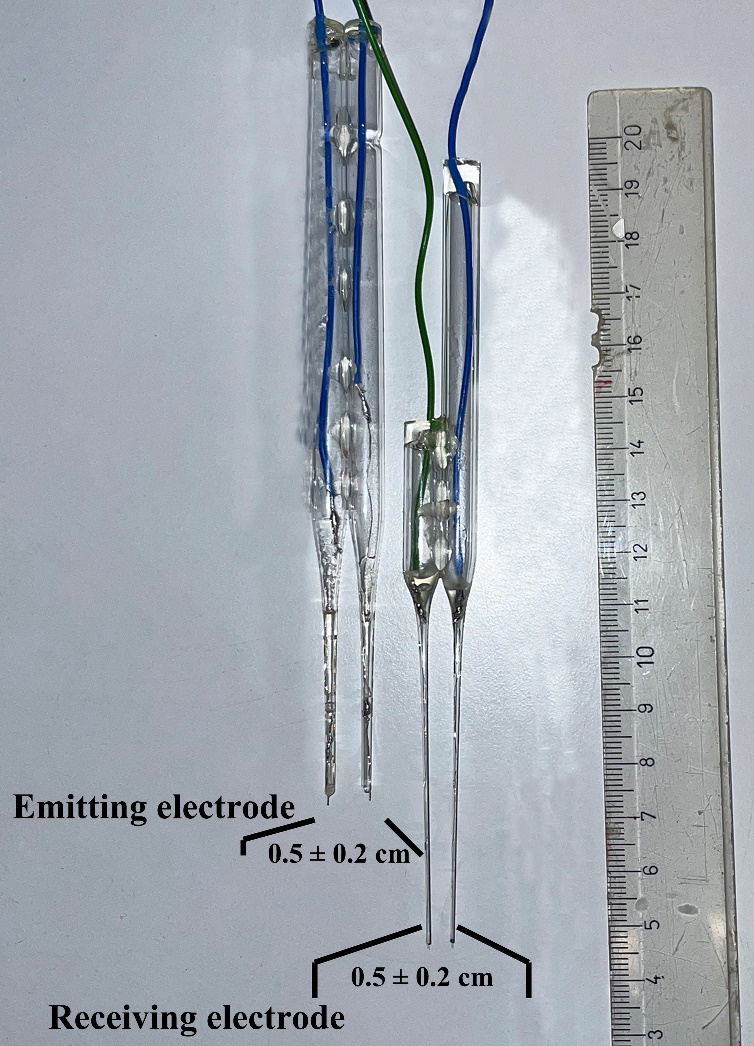
**

**Extended Data Fig. S2**. Emitting electrode and receiving electrode for this study. The length of the Pt tips of the receiving electrode is about 250 μm, and the diameter of the Pt wires is 50 μm (see the Extended Data Fig. S3.). Except for the exposed Pt tips, the other part of the Pt wires is coated by glass with an outer diameter of 5 mm (Schott 8366; wall thickness: 0.5 mm). The Pt wires is drawn out by cable and connected to the biological signal acquisition and processing system (MedLab®-U/4C501H, China).

**
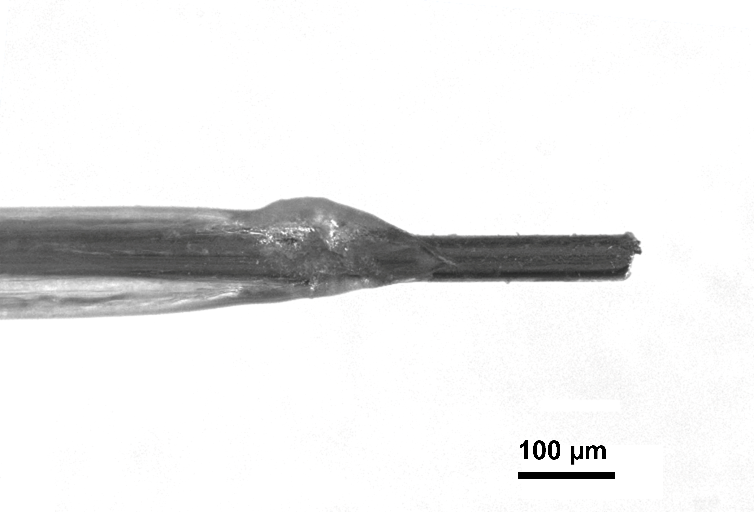
**

**Extended Data Fig. S3.** The naked Pt tips of the emitting electrode and receiving electrode. The naked Pt tips for the receiving electrode (250 μm) are purposed to contact the cable bacterial filaments and thus are very important for this measurement of induced signals.

**
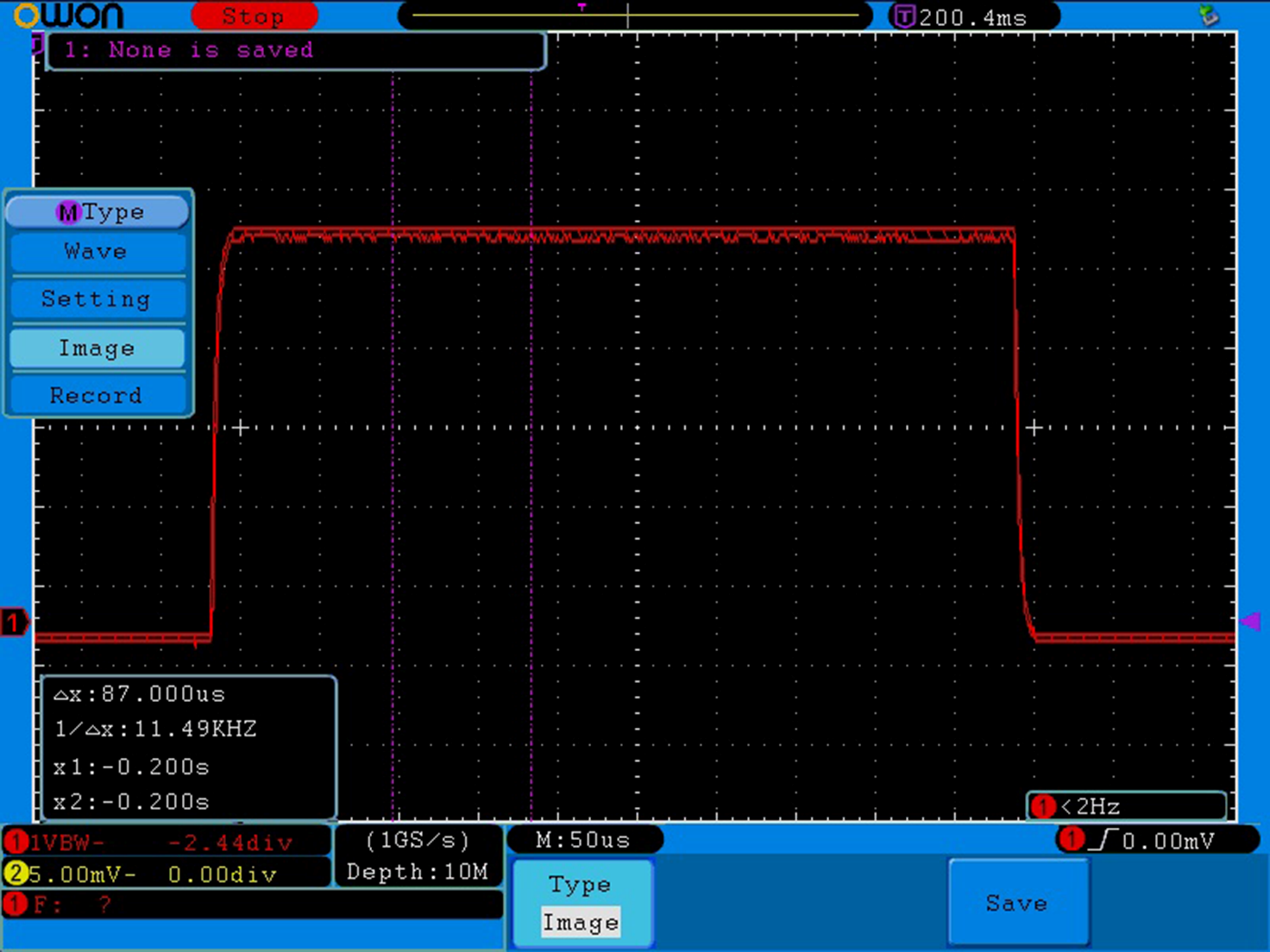
**

**Extended Data Fig. S4**. Time-varying electric field stimulus with a square wave between two naked Pt tips. The square wave, characterized by an oscilloscope (DS2102, OWON, China), presents a 5.0-mV wave height. In figures 1-4 of the paper, all wave parameters are the same except for the difference in wave width.

**

**

**Extended Data Fig. S5**. Relationship between the width of square waves and time interval of induction signals in cable bacterial layer. The time difference between two induced signals is always equal to the width of the applied square waves because the induction accompanies the formation and the disappearance of the applied square wave radio (also called electromagnetic oscillation). This slope (1.00) for linear correlation shows confirmed the dependence of induced signals on electromagnetic oscillation.

**
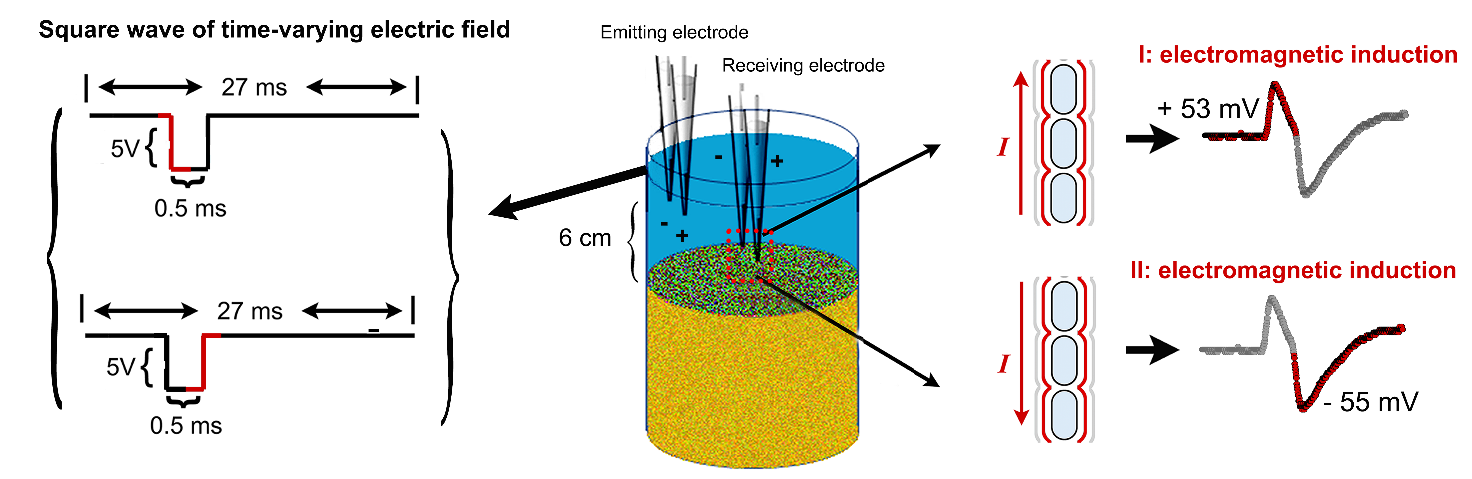
**

**Extended Data Fig. S6**. Square waveform voltage from -5.0 to 0 V vs. induction signals in cable bacterial layer. Compared to Figure 2, a negative square wave was applied to between two naked Pt tips of the emitting electrode, which means that the rate of change of the spatial electromagnetic field is completely opposite to that of Figure 2A and B (left), and thus correspondingly produces two completely opposite inductive signals (comparing the right of Extended Data Fig. S6 with the right of Figure 2A and B).

**
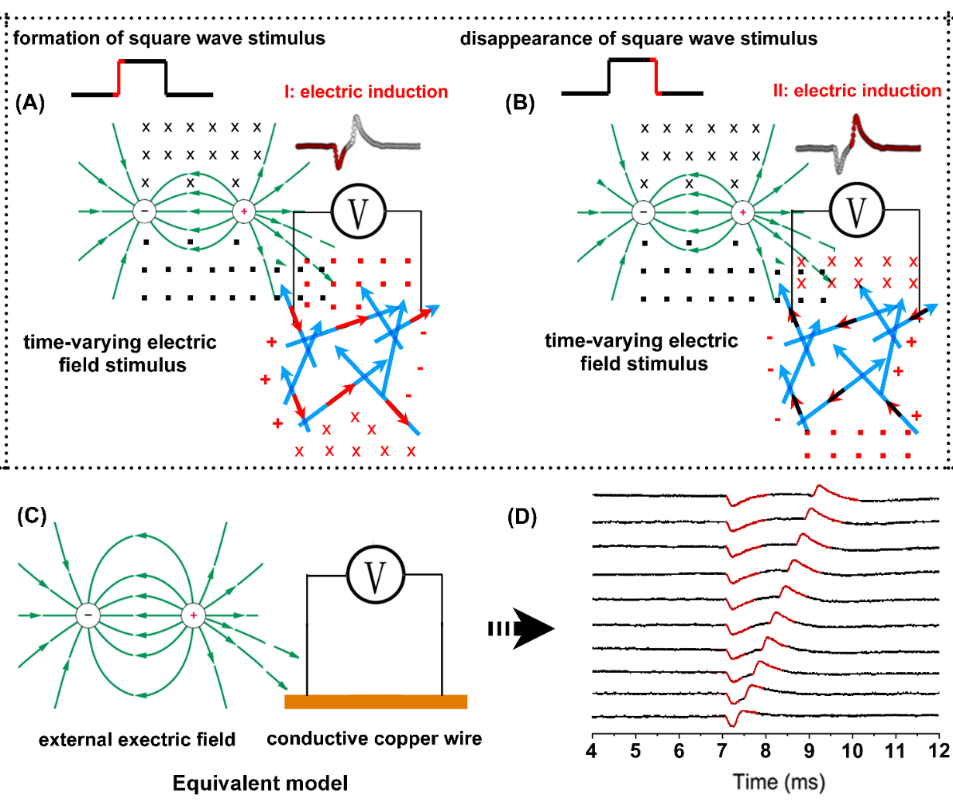
**

**Extended Data Fig. S7.** The proposed equivalent model for electromagnetic induction in filamentous cable bacteria. (**A**): An increase in a periodic square-wave voltage accompanied by the formation of square waves causes an induced magnetic field, which induces filamentous bacteria to produce a reverse electrical signal. The green contour lines represent external electric fields applied to seawater (produced by square waves of the time-varying electric field stimulus; the same below). Black cross: The magnetic field from the square wave is perpendicular to the plane, and the direction is inward (the same below); black dot: The magnetic field from the square wave is perpendicular to the plane, and the direction is outward (the same below). The blue lines represent the filamentous cable bacteria in sediment, and the arrows show the physiological electron migration along bacteria from anaerobic to aerobic regions of sediment. This electron migration is only to satisfy the physiological activity of cable bacteria and has nothing to do with the electronic activity under the action of the external electric field. Red cross: The magnetic field from the induced filamentous bacteria is perpendicular to the plane, and the direction is inward (the same as below); Red dot: The magnetic field from the induced filamentous bacteria is perpendicular to the plane, and the direction is outward (the same below). (**B**): Decrease in a periodic square-wave voltage accompanied by the disappearance of square waves also causes an induced magnetic field, which induces filamentous bacteria to generate a positive electrical signal. (**C**): The proposed equivalent model of bio-induction. Here, we used a 1-cm long copper wire (diameter = 0.2 mm) to replace the filamentous cable bacteria in **Extended Data Fig. S7**A or B, and other test conditions were exactly the same as in **Extended Data Fig. S7**A or B. (**D**): Negative and positive induction signals produced by the equivalent model of bio-induction, with the different time-varying electric field stimulus (amplitude: 5.0 V from 0.2, 0.4, 0.6, 0.8, 1.0, 1.2, 1.4, 1.6, 1.8, and 2.0 ms). The periodic square waves that produce the different time-varying electric fields are the same as those on the left of Fig. 2C.


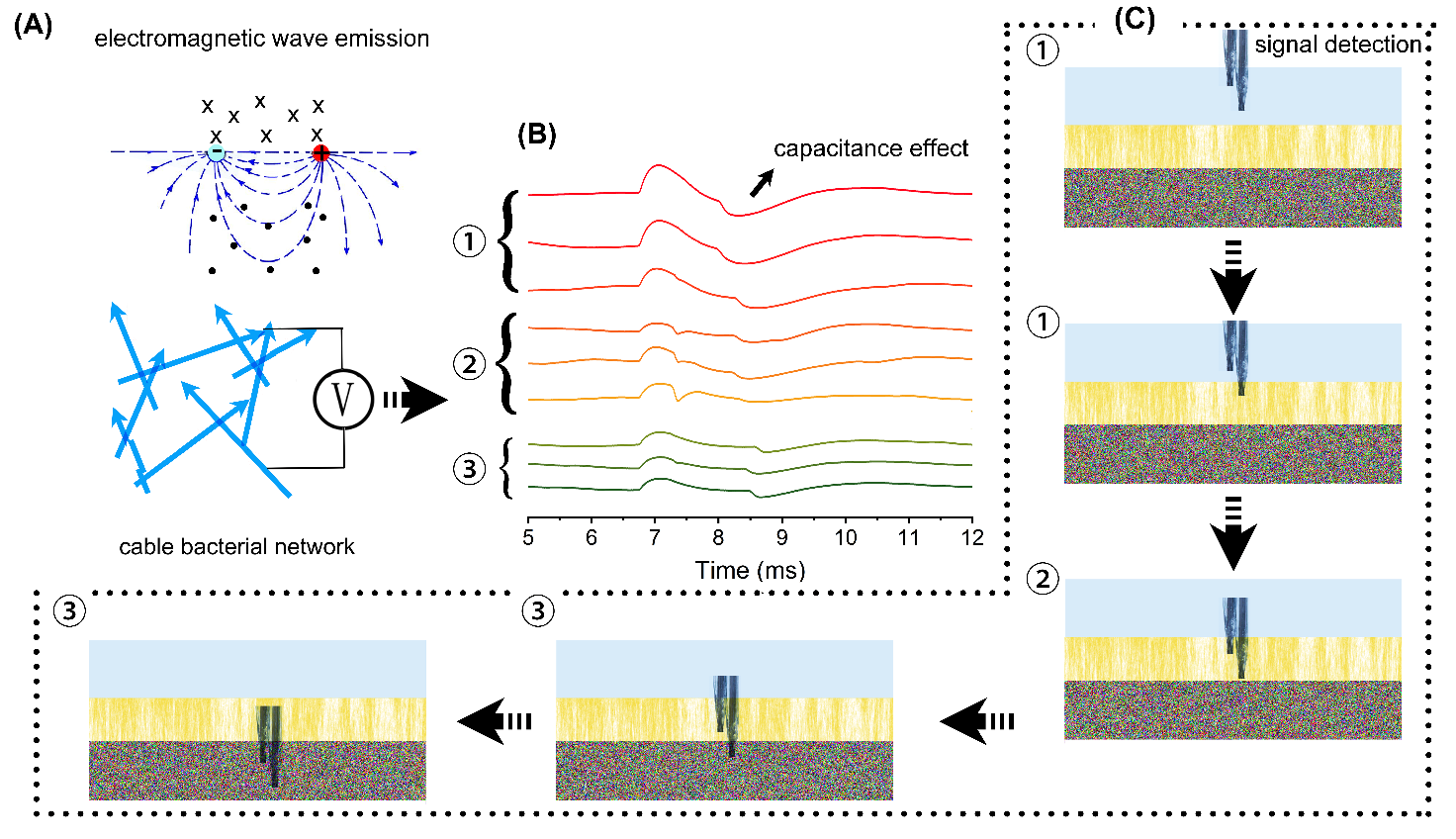


**Extended Data Fig. S8.** Vertical component of bio-induction in cable bacterial layer of sediment. (**A**)**:** The principle of detection for this study. The arrangement of the emitting electrode is the same as Fig. 1A. (**B**): Vertical component of the signals stimulated by the emitting electrode. (**C**): a receiving electrode at different detection positions. Light blue: seawater; light yellow: cable bacterial layer; black mélange: deeper mineral sediment. The receiving electrode with an upper and lower tip spacing of 1cm is used to detect the vertical component of the signals induced by the emitting electrode. The horizontal spacing between the two poles of the emitting electrode is 1.5 mm. The test was performed under cable bacteria with (42–46) per square millimeter (under microscopic examination).
